# Human infection with *Plasmodium knowlesi* on the Laos-Vietnam border

**DOI:** 10.1186/s41182-018-0116-7

**Published:** 2018-09-18

**Authors:** Tiengkham Pongvongsa, Richard Culleton, Hoang Ha, Le Thanh, Panom Phongmany, Ron P. Marchand, Satoru Kawai, Kazuhiko Moji, Shusuke Nakazawa, Yoshimasa Maeno

**Affiliations:** 1 Savannakhet Provincial Health Department, Savannakhet, Laos; 2 Malaria Unit, Department of Pathology, Institute of Tropical Medicine, Nagasaki University, Nagasaki, Nagasaki Japan; 3 Faculty of Public Health, Da Nang University of Medical Technology and Pharmacy, Da Nang, Vietnam; 4 Malaria-Parasitology and Entomology Department, Preventive Medicine Center of Quang Tri province, Quang Tri province, Vietnam; 5 Khanh Phu Malaria Research Unit, Medical Committee Netherlands-Viet Nam, Nha Trang, Khanh Hoa Province Vietnam; 6 Laboratory of Tropical Medicine and Parasitology, Dokkyo Medical University, Mibu, Tochigi, Japan; 7 Graduate School of Tropical Medicine & Global Health, Nagasaki University, Nagasaki, Nagasaki Japan; 8 Department of Protozoology, Institute of Tropical Medicine, Nagasaki University, Nagasaki, Nagasaki Japan; 9 Department of Virology and Parasitology, Fujita Health University School of Medicine, 1-98 Kutsukake, Toyoake, Aichi 470-1192 Japan

**Keywords:** *Plasmodium knowlesi*, Border malaria, Laos, Vietnam, Greater Mekong region, Forest, Monkeys, Human

## Abstract

**Background:**

Border malaria in the Greater Mekong region of Southeast Asia poses a serious threat to the health of the ethnic minority populations of the region. Traditionally thought to be caused primarily by the malaria parasites *Plasmodium falciparum* and *Plasmodium vivax*, recently a zoonotic parasite, *Plasmodium knowlesi*, has been identified in some countries of the region. The presence of this parasite poses a challenge to malaria control programmes, as it is maintained in a zoonotic reservoir of forest-dwelling macaque monkeys.

**Methods:**

A cross-sectional malaria parasite species prevalence survey was conducted along the Laos-Vietnam border in the central part of the two countries. Human blood samples were collected from Savannakhet in Laos and Quang Tri in Vietnam between August and October 2010 and assayed for the presence of human malaria parasite species and *P. knowlesi.* A PCR targeting the 18S small subunit ribosomal RNA gene and circumsporozoite protein gene was used for *Plasmodium* species identification.

**Results:**

Nine cases of *P. knowlesi* were detected by PCR in blood samples from the Laos side and three from the Vietnam side. All *P. knowlesi* infections were found in co-infection with *P. vivax*, with some triple infections of *P. knowlesi*, *P. vivax* and *P. falciparum* detected in Laos. Phylogenetic analysis of these parasites suggests that *P. knowlesi* is circulating in the Laos-Vietnam border region.

**Conclusion:**

This report shows that *P. knowlesi* is transmited on both sides of the Vietnam-Laos border. Continued monitoring of the range and prevalence of *P. knowlesi* on both the sides of Laos-Vietnam border is of importance to the National Malaria Control Programmes of both countries.

**Electronic supplementary material:**

The online version of this article (10.1186/s41182-018-0116-7) contains supplementary material, which is available to authorized users.

## Background

Malaria transmission in the Greater Mekong region of Southeast Asia is characterised by the spread of the parasite among people residing near, or travelling across, the numerous borders between the six countries of the region in which malaria is endemic. Often termed “border malaria”, it is characterised by transmission among and between diverse, often forest dwelling, and ethnic minority groups and may have a sylvatic component. Recently, numerous reports have documented the occurrence of infections among the human population in this region of the zoonotic malaria species, *Plasmodium knowlesi*, a parasite that naturally infects the Cercopithecinae Old World monkey species *Macaca fascicularis*, and *Macaca nemestrina* [1–5]. It is possible that the parasite is transmitted to humans in this region through the bites of the leucosphyrus group of anopheline mosquitoes, known vectors of both human and non-human primate malaria [6–11].

The heavily forested border area between Laos and Vietnam is home to various ethnic minority peoples [12]. These peoples engage in many forest activities as part of their way of life, mainly swidden agriculture (slash-and-burn farming), hunting and foraging. Frequent and prolonged forays into forested areas increase the risk of sylvatic and zoonotic malaria infections among these groups. In Laos, a recent case of *P. knowlesi* in a human has been reported from Attapeu province, near the Cambodia border [13]. However, the extent of the risk posed by *P. knowlesi* to humans in this region has not been addressed. The neighbouring country of Vietnam has previously reported the infection of people with *P. knowlesi* parasites [3], and, as the border area between Laos and Vietnam may act as a conduit for the transmission of malaria parasites between groups of people, monkeys and mosquitoes, it is conceivable that the parasite might be circulating in the border region, and so may pose a risk to the population of Laos.

## Materials and methods

A cross-sectional survey for malaria infection was conducted between Savannakhet, Laos and Quang Tri, Vietnam, between August and October 2010 in order to evaluate malaria parasite prevalence along the border [12]. Detailed information regarding study sites, populations surveyed, methodology of sample collection and ethics review have been published elsewhere [12]. All adult volunteers provided informed consent and for children, and consent was obtained from close relatives. Human sample analysis was approved by the ethics committee of the Institute of Tropical Medicine, Nagasaki University (10121662-5). Active case detection (ACD) was conducted during the survey in 400 randomly selected households from 22 villages on the Vietnam side and from 14 villages on the Laos side. The Laotian and Vietnamese villages are separated by a shallow river around 50-m wide and are surrounded by forested mountains. A small number of villagers keep young macaque monkeys caught in the nearby forest as pets. The people that live in this region, on both sides of the border, are a minority ethnic group known as the Van Kieu in Vietnamese [14] and the Tri in Lao, who have special permission from the governments of Laos and Vietnam to freely cross the border.

The samples used in this study were collected as previously described [12]. A total of 3059 samples (Laos, 1256; Vietnam, 1803) were analysed by microscopy for the presence of malaria parasites (Table 1). Questionnaires were used to collect information such as addresses, medical examination results and treatment records, and blood was collected by finger-prick; thick blood films were made for microscopy, and blood was applied to filter paper for molecular analyses. Blood smears were examined by local microscopists at the provincial malaria laboratories of each country and then crosschecked. Each blood-spotted filter paper was immediately air dried, placed in a sealed plastic bag and stored at room temperature. All malaria cases were treated as per the national guidelines for malaria treatment of each country.

**Table 1:** Number of samples for this study and results of microscopic examination [12]

	Laos	Vietnam
No. of blood examinations	1256	1803
No. of *Plasmodium* spp. positive by microscopy	63	33
Detected *Plasmodium* species by microscopy		
*P. falciparum*	30	18
*P. vivax*	32	14
*P. falciparum* + *P. vivax*	1	1
No. of microscopy positive by age group		
≤ 5 years	12	5
6–14 years	33	12
≥ 15 years	18	16
No. of microscopy positive by gender		
Male	30	18
Female	33	15

A of total 135 samples (Laos, 110; Vietnam, 25) were used for PCR analysis (Table 2). DNA was extracted from dried blood samples on filter paper, and subsequent PCR analysis was carried out as previously described [14, 15]. Briefly, each dried blood spot was cut into small pieces by a razor blade. The razor blade was cleaned with alcohol wipes and flamed at every sample cut to prevent contamination and to inactivate DNase. gDNA was extracted using a QIAamp DNA micro kit (QIAGEN, Tokyo, Japan). *Plasmodium* species-specific nested PCR assays to identify human, and *P. knowlesi* were performed as described [10]. The genus-specific primers, rPLU-1/rPLU-5, were used in the primary amplification (nest 1) and performed as described by Singh et al. (1999) [15]. Detection of species-specific 18S rRNA genes (nest 2) was performed as previously described [5, 15, 16]. For the nest 2, 2 μL of × 50 nest 1 amplification product was used as the template in the reaction mixtures (25 μL). PCR products were separated by electrophoresis on 1.5% agarose gels and stained with ethidium bromide. DNA bands were analysed with Lane & Spot Analyzer software (Atto, Tokyo, Japan).

**Table 2:** Summary *Plasmodium* species of human blood collected Laos and Vietnam by PCR analysis

	Laos		Vietnam
	Microscopy (+)	Microscopy (−)	Microscopy (+)
No. of examined by PCR	35	75	25
No. of *Plasmodium* spp. positive by PCR	35	30	25
Detected *Plasmodium* species by PCR			
*P. falciparum*	21	12	16
*P. vivax*	0	9	2
*P. falciparum* + *P. vivax*	5	6	4
*P. falciparum + P. malariae*	2	0	0
*P. vivax + P. knowlesi*	1	1	3
*P. falciparum* + *P. vivax + P. malariae*	1	0	0
*P. falciparum* + *P. vivax* + *P. knowlesi*	5	2	0
No. of PCR positive by age group			
≤ 5 years	10	8	4
6–14 years	14	9	5
≥ 15 years	11	13	16
No. of PCR positive by gender			
Male	16	10	14
Female	19	20	11

For the detection of the *P. knowlesi* 18S rRNA gene, two kinds of primer sets, pmk8/pmk9 [16] and Kn1f/Kn3r [5], were used. It is known that the primer set Pmk8/Pmk9 can occasionally cross-react with *P. vivax* DNA and produce false-positive results whereas primer set Kn1f /Kn3r does not (Additional file 1). For confirmation of *P. knowlesi* infection, detection of the *P. knowlesi* circumsporozoite protein (CSP) gene was carried out as previously described by Vythilingam et al. (2008) [17]. Primer sequences for *18S rRNA* of human malaria parasites and *P. knowlesi*-specific primers and the CSP gene of *P. knowlesi* were as previously described [5, 15–17]. *P. knowlesi* H strain DNA (American Type Culture Collection no. 30158) was used as a positive control. We verified that no cross-reaction occurred between the primer sets used to amplify *P. vivax* CSP and *P. knowlesi* CSP by using DNA extracted from single infections of both species (data not shown). A sample was only considered positive for *P. knowlesi* if all three assays (pmk8/pmk9, Kn1f/Kn3r and PkCSP) gave a positive result.

For nucleotide sequencing, the specific products resulting from PCR amplification of the CSP and 18S rRNA genes were cleaned using the Wizard SV Gel and PCR Clean-up System (Promega, Tokyo, Japan) according to the manufacturer’s instructions and were then sequenced with the BigDye Terminator v3.1 Cycle Sequencing Premix Kit (Applied Biosystems, Inc.). The reaction products for sequencing were separated with an ABI/Hitachi 3130 × 1 Genetic Analyzer (ABI), and the resulting nucleotide sequences were compiled using Genetyx (Genetyx Corporation, Tokyo, Japan).

## Results and discussion

In Laos, 1256 persons were recruited for microscopic blood examination, of which 63 (5%) were positive for malaria parasites, 30 for *Plasmodium falciparum* and 32 for *Plasmodium vivax* with one mixed infection (*P. falciparum* + *P. vivax*) (Table 1). Parasite prevalence was highest in the southern part of the study area, and children under 15 years old were significantly more likely to carry parasites than adults. Of 63 malaria positive cases, 35 samples were available for molecular screening of *P. knowlesi* and human malaria parasites (Table 2). In order to thoroughly screen for *P. knowlesi*, 75 slide-negative samples collected from family members of microscopy positive patients were also screened. *Plasmodium knowlesi* infection was assayed using three PCRs, two based on the 18S rRNA gene and one on the CSP gene. Only samples positive for all three assays were considered to be *P. knowlesi* positive (Additional file 1). Nine out of 110 samples were positive for *P. knowlesi* (Table 2). Six were detected among the 35 microscopic positive samples (17%) and three cases among the three microscopic negative samples (3%). *Plasmodium knowlesi* was exclusively detected in samples collected from children aged 2 to 10 years old and was always found in co-infection with either *P. falciparum* or *P. vivax* (Table 3). In two cases, we observed *P. knowlesi* infection of two family members simultaneously: cases 3 and 4, and cases 8 and 9 were infections of brothers in the same families (Table 3).

**Table 3:** Characteristics of people infected with *Plasmodium knowlesi*

Country	Case	Age	Family no.	Gender	Microscopy	Parasites by PCR	Temperature (°C)
Laos	1	2	11020	Male	(−)	Pk + Pv	38.4
	2	5	13008	Female	Pv	Pk + Pv	36.0
	3	5	14013	Female	Pf	Pk + Pv + Pf	36.5
	4	5	14013	Male	(−)	Pk + Pv + Pf	35.8
	5	5	14014	Female	(−)	Pk + Pv + Pf	37.8
	6	5	14017	Female	Pv	Pk + Pv + Pf	36.5
	7	7	14108	Male	Pf	Pk + Pv + Pf	37.0
	8	8	14034	Male	Pv	Pk + Pv + Pf	37.0
	9	10	14034	Male	Pv	Pk + Pv + Pf	36.5
Vietnam	1	4	N/A	Male	Pv	Pk + Pv	N/A
	2	11	N/A	Male	Pv	Pk + Pv	N/A
	3	15	N/A	Female	Pv	Pk + Pv	N/A

*Pv Plasmodium vivax*, *Pk P. knowlesi*, *Pf P. falciparum*, *N/A* not available

In Vietnam, of 1803 persons recruited for microscopic blood examination, and 35 (2%) were positive for malaria parasites. Of these, 18 were infected with *P. falciparum*, 14 with *P. vivax*, and one with a *P. falciparum* + *P. vivax* mixed infection (Table 1). Malaria prevalence was highest in the southern villages, as in Laos. Of the 35 microscopic positive cases, 25 blood samples were available for PCR analysis for the identification of *Plasmodium* spp. Three out of these 25 samples (12%) were positive for *P. knowlesi*, all in co-infections with *P. vivax* (Table 2).

**Fig. 1:**
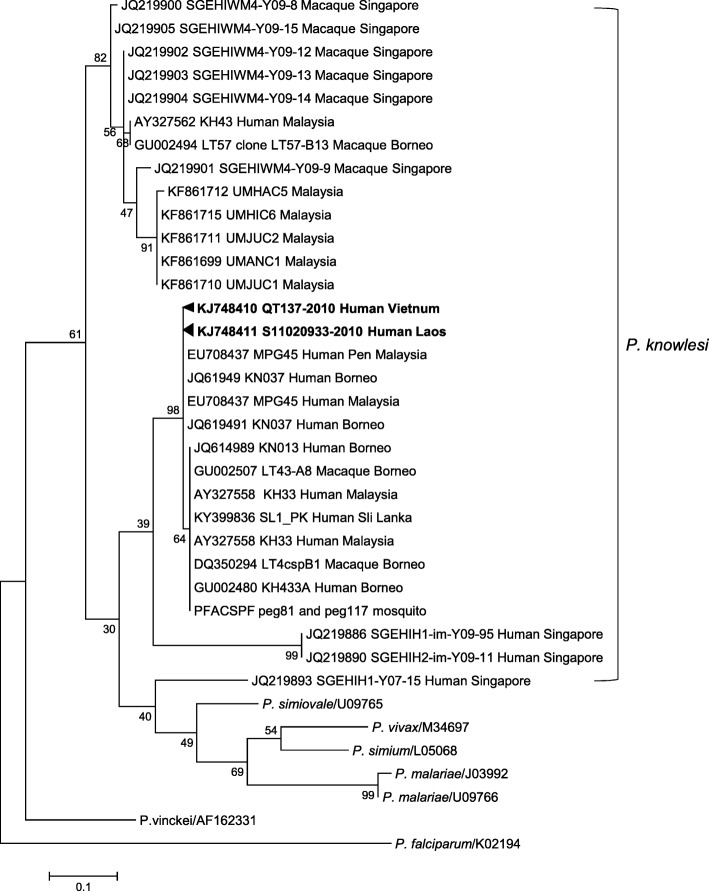
Phylogenetic tree constructed from the nucleotide sequences of the CSP gene of *Plasmodium* spp*.* The evolutionary history was inferred by using the maximum likelihood method based on the Tamura-Nei model. Number of substitutions per site is indicated by the scale bar. Bootstrap values were calculated for 1000 replications. Phylogenetic analysis was conducted using MEGA7. Scale bar indicates nucleotide substitutions per site

Molecular diagnosis of *P. knowlesi* was performed by the independent amplification of two genes (*18S rRNA* and *csp*), and PCR products were sequenced for confirmation of diagnosis. This molecular diagnosis was carried out independently at three separate institutions. Twelve out of 135 samples examined by PCR were confirmed as *P. knowlesi* through sequencing of the CSP and 18S rRNA gene (Fig. 1 and Additional file 2). All the *P. knowlesi* parasites detected in this study were found in co-infections with *P. vivax*, either in dual-species infections or (but only in Laos) triple-species infections along with *P. falciparum* and *P. vivax* (Table 2). We did not detect any dual infections of *P. knowlesi* with *P. falciparum* (Table 2), and similar findings have been previously reported in Vietnam [3]. No single *P. knowlesi* infections were found in this study, although a single *P. knowlesi* infection case was recently reported from Attapeu province (adjacent to Cambodia), Laos [13].

All the *P. knowlesi* infections in this study were detected in children under 15 years old. In contrast, infection with human malaria parasites often occurred in people older than 15 (Tables 2 and 3), and a similar tendency was reported in Southern Vietnam [3]. This may be a consequence of age-related acquired immunity against the parasites, as well as the fact that our analysis was skewed towards children [12]. The body temperature of children carrying *P. knowlesi* was normal except cases 1 and 5 (Table 3), although the cyclical nature of malarial fever means that the timing of temperature monitoring is crucial, and it is possible that fever episodes in these individuals were missed. The asymptomatic presentation of *P. knowlesi* infection described here differs from the symptomatic infections observed in Malaysia, where individuals infected with *P. knowlesi* suffer malaria disease and occasionally develop severe illness [18]. It is possible that this difference is a consequence of the fact that *P. knowlesi* was only found in co-infections with *P. vivax* in our study. Other factors that could contribute to this difference include the genetics of the parasites and their hosts, and transmission intensities.

It is known that *M. fascucularis* and *M. nemestrina* are natural hosts of multiple species of non-human primate malaria parasites [5, 19–21]. These macaques are present in the study area [20, 21] where *Anopheles dirus* and *Anopheles minimus*, known vectors of *P. knowlesi* [3, 10, 11, 22], have previously been captured [12]. The presence of monkey reservoirs and competent mosquito vectors is consistent with the hypothesis that *P. knowlesi* infection of humans here is a zoonotic phenomenon, although human-human transmission cannot be excluded.

## Conclusions

This molecular epidemiological study describes the presence of multiple natural human infections of *P. knowlesi* in Laos. We also report that *P. knowlesi* is transmitted on both sides of the Vietnam-Laos border—although the villages are so near to each other that it would have been odd if it occurred only the one side of the river. Although *P. knowlesi* has not been widely reported in Southeast Asia, it has the potential to cause severe disease in humans, and so, its prevalence should be closely monitored [18]. Furthermore, as humans continue to encroach into previously uninhabited regions, and contact between monkey, mosquito and human populations becomes more frequent, *P. knowlesi* and, potentially, other macaque malaria parasite species, have the potential to become more prevalent in the human population. Therefore, continued monitoring of the range and prevalence of *P. knowlesi* is of importance to the National Malaria Control Programmes of both countries.

## Additional files

Additional file 1: Agarose gel (1.5%) electrophoresis of PCR products for the identification of *Plasmodium* species using DNA extracted from dried blood on the filter paper. M, 100 bp size marker; QT65, QT250, QT835, XP677; human dried blood samples; NC, negative control; Pv (VIV1/VIV2), 18S rRNA of *Plasmodium vivax*; Pk (Pmk8/Pmk9) and Pk (knf1/knf3), 18S rRNA of *Plasmodium knowlesi*; Pk CSP, CSP of *Plasmodium knowlesi*. (PDF 649 kb)

Additional file 2: Phylogenetic tree constructed from the nucleotide sequences of the 18S rRNA gene of *Plasmodium* spp*.* The evolutionary history was inferred by using the maximum likelihood method based on the Tamura-Nei model. Number of substitutions per site is indicated by the scale bar. Bootstrap values were calculated for 1000 replications. Phylogenetic analysis was conducted by using MEGA7. Scale bar indicates nucleotide substitutions per site. S-2010 rRNA gene Human Laos are representative Laos specimen. QT-2010 rRNA gene Human Vietnam are representative Vietnam specimen. (PDF 649 kb)
