# Supplementary material for: Human infection with Plasmodium knowlesi on the Laos-Vietnam border
Source: Trop Med Health. 2018 Sep 18;46:33. doi: 10.1186/s41182-018-0116-7 (PMC6145087; doi:10.1186/s41182-018-0116-7)
Supplement: Supplementary file 1 — Agarose gel (1.5%) electrophoresis of PCR products for the identification of Plasmodium species using DNA extracted from dried blood on the filter paper. M, 100 bp size marker; QT65, QT250, QT835, XP677; human dried blood samples; NC, negative control; Pv (VIV1/VIV2), 18S rRNA of Plasmodium vivax; Pk (Pmk8/Pmk9) and Pk (knf1/knf3), 18S rRNA of Plasmodium knowlesi; Pk CSP, CSP of Plasmodium knowlesi. (PDF 649 kb) [file 41182_2018_116_MOESM1_ESM.pdf]

Additional file1. Tiengkham et al.

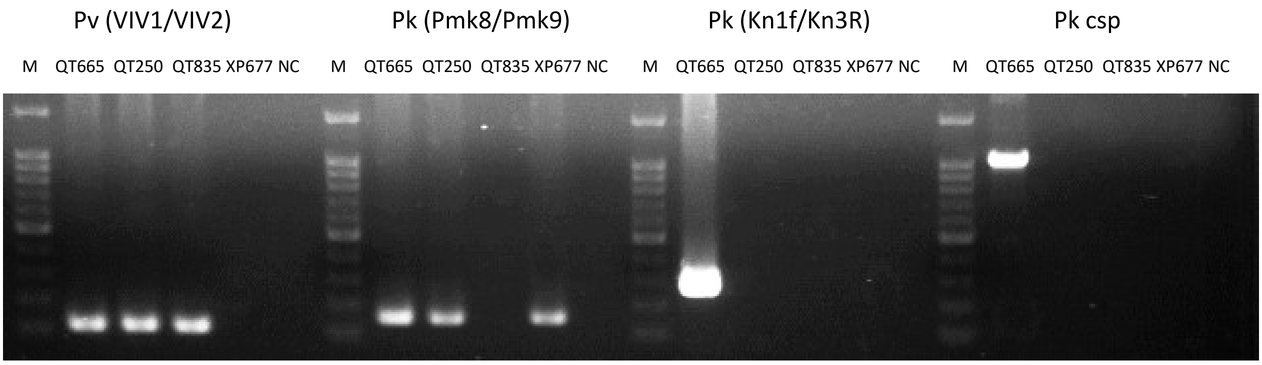

Additional file1. Agarose gel (1.5%) electrophoresis of PCR products for the identification of *Plasmodium* species using DNA extracted from dried blood on the filter paper. M, 100 bp size marker; QT65, QT250, QT835, XP677; human dried blood samples; NC, negative control; Pv (VIV1/VIV2), 18S rRNA of *Plasmodium vivax*; Pk (Pmk8/Pmk9) and Pk (knf1/knf3), 18S rRNA of *Plasmodium knowlesi*; Pk CSP, CSP of *Plasmodium knowlesi*.
