# Supplementary material for: Human infection with Plasmodium knowlesi on the Laos-Vietnam border
Source: Trop Med Health. 2018 Sep 18;46:33. doi: 10.1186/s41182-018-0116-7 (PMC6145087; doi:10.1186/s41182-018-0116-7)
Supplement: Supplementary file 2 — Phylogenetic tree constructed from the nucleotide sequences of the 18S rRNA gene of Plasmodium spp. The evolutionary history was inferred by using the maximum likelihood method based on the Tamura-Nei model. Number of substitutions per site is indicated by the scale bar. Bootstrap values were calculated for 1000 replications. Phylogenetic analysis was conducted by using MEGA7. Scale bar indicates nucleotide substitutions per site. S-2010 rRNA gene Human Laos are representative Laos specimen. QT-2010 rRNA gene Human Vietnam are representative Vietnam specimen. (PDF 649 kb) [file 41182_2018_116_MOESM2_ESM.pdf]

Additional file 2. Tiengkham et al.

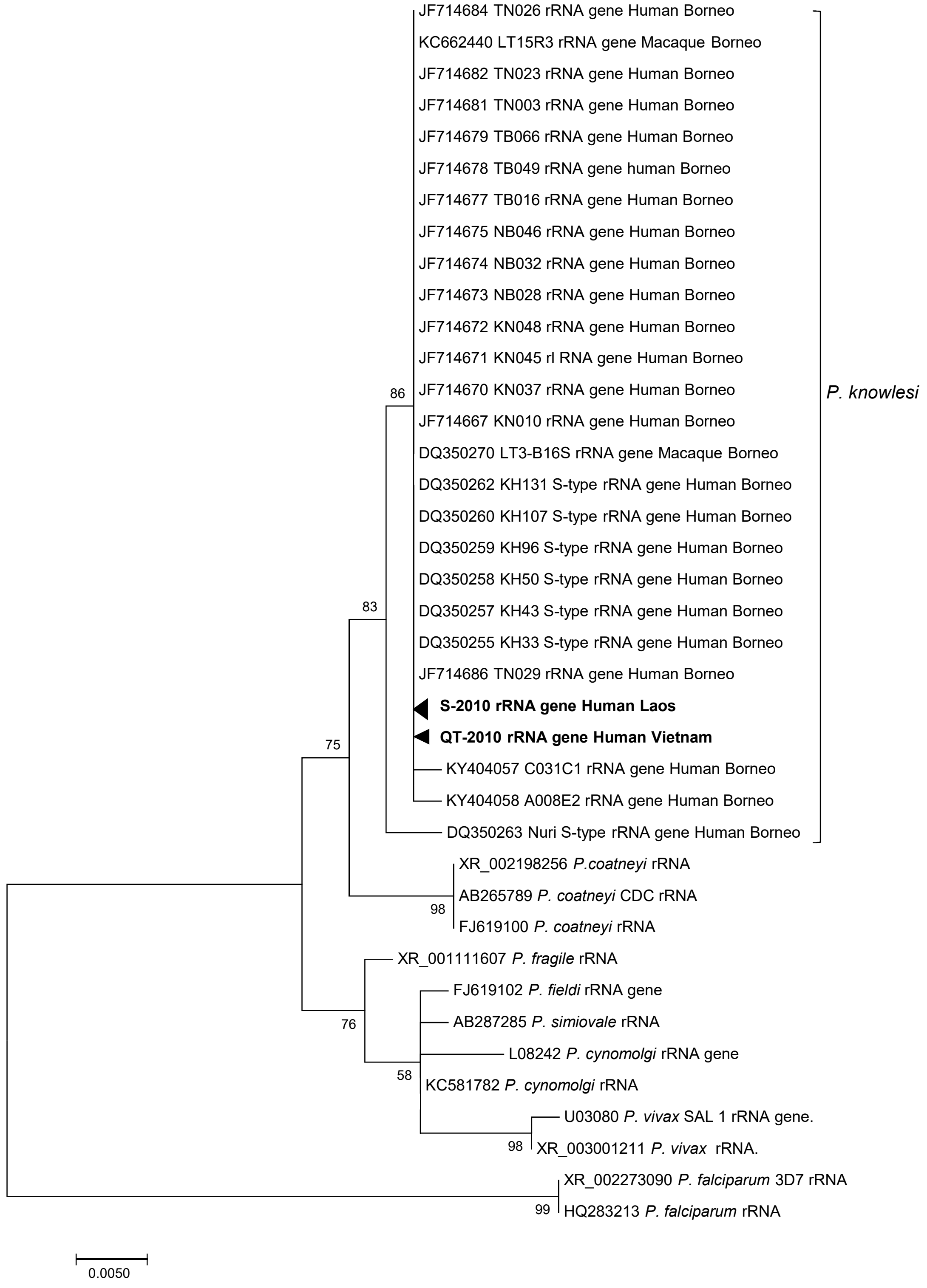

Additional file2. Phylogenetic tree constructed from the nucleotide sequences of the 18S rRNA gene of *Plasmodium* spp. The evolutionary history was inferred by using the Maximum Likelihood method based on the Tamura-Nei model. Number of substitutions per site is indicated by the scale bar. Bootstrap values were calculated for 1000 replications. Phylogenetic analysis was conducted by using MEGA7. Scale bar indicates nucleotide substitutions per site. S-2010 rRNA gene Human Laos are representative Laos specimen. QT-2010 rRNA gene Human Vietnam are representative Vietnam specimen.
